# A Review of Fundamental Optimization Approaches and the Role of AI Enabling Technologies in Physical Layer Security

**DOI:** 10.3390/s22093589

**Published:** 2022-05-09

**Authors:** Mulugeta Kassaw Tefera, Zengwang Jin, Shengbing Zhang

**Affiliations:** School of Cyberspace Security, Northwestern Polytechnical University, 127 West Youyi Road, Xi’an 710072, China; mulugeta@nwpu.edu.cn (M.K.T.); jin_zengwang@nwpu.edu.cn (Z.J.)

**Keywords:** physical layer security, optimization approaches, information theory, signal processing techniques, resource allocation, AI techniques

## Abstract

With the proliferation of 5G mobile networks within next-generation wireless communication, the design and optimization of 5G networks are progressing in the direction of improving the physical layer security (PLS) paradigm. This phenomenon is due to the fact that traditional methods for the network optimization of PLS fail to adapt new features, technologies, and resource management to diversified demand applications. To improve these methods, future 5G and beyond 5G (B5G) networks will need to rely on new enabling technologies. Therefore, approaches for PLS design and optimization that are based on artificial intelligence (AI) and machine learning (ML) have been corroborated to outperform traditional security technologies. This will allow future 5G networks to be more intelligent and robust in order to significantly improve the performance of system design over traditional security methods. With the objective of advancing future PLS research, this review paper presents an elaborate discussion on the design and optimization approaches of wireless PLS techniques. In particular, we focus on both signal processing and information-theoretic security approaches to investigate the optimization techniques and system designs of PLS strategies. The review begins with the fundamental concepts that are associated with PLS, including a discussion on conventional cryptographic techniques and wiretap channel models. We then move on to discuss the performance metrics and basic optimization schemes that are typically adopted in PLS design strategies. The research directions for secure system designs and optimization problems are then reviewed in terms of signal processing, resource allocation and node/antenna selection. Thereafter, the applications of AI and ML technologies in the optimization and design of PLS systems are discussed. In this context, the ML- and AI-based solutions that pertain to end-to-end physical layer joint optimization, secure resource allocation and signal processing methods are presented. We finally conclude with discussions on future trends and technical challenges that are related to the topics of PLS system design and the benefits of AI technologies.

## 1. Introduction

Currently, 5G wireless networks are fully commercialized worldwide and B5G networks are in the process of development and are supposed to be deployed within the next few years. With the fast adoption of 5G technology, the number of users who utilize wireless mobile networks has increased exponentially over the last few years. It is expected that this rapid growth will continue to increase enormously due to the deployment of more smart applications and new enabling technologies within the upcoming B5G networks. Future wireless networks will not only provide much higher spectral efficiency/data rates and lower latency but will also provide new services and technologies that can be applied in various vertical industries [1]. The presence of ubiquitous connections and the growing number of devices that are connected to the internet are expected to cause a challenge for efficient and reliable resource management systems [2,3,4]. Moreover, the rapid growth of the massive number of new devices that are connected to the internet and the Internet of Things (IoT) is expected to cause a serious risk to network security if not manipulated properly [5]. Therefore, when considering all of these capabilities, it can be seen that there is a need for robust security mechanisms across all segments of 5G and B5G networks.

Conventionally, high-layer cryptography-based techniques have been widely adopted to deal with any discrepancies that are associated with information confidentiality, which include data authentication, secret key establishment and secret dissemination [6]. However, with the advancement in the computing capacities of eavesdropping devices, the above-mentioned techniques may not be sufficient or may even become unsuitable when an extra secure channel is required for secret key generation [7]. Recently, physical layer security (PLS) has emerged as a promising means of addressing the eavesdropping computational capability of secure transmission problems [8,9,10,11,12]. Compared to complex cryptography techniques, PLS does not depend on the computational capacity of the eavesdropping devices and, therefore, it has the benefit of reducing computational costs and resource consumption. From the perspective of information-theoretic fundamentals, it has been found that PLS can achieve secure and reliable communication even when network intruders have very strong computing capabilities [13]. This approach to information security is especially effective since it does not rely on underlying computational capabilities, but rather on the characteristics of the transmission media, such as noise, fading and interference, and it provides security guarantees that are independent from the computing power of the eavesdropper. In general, the PLS approach presents distinct advantages and is well suited for distributed processing systems and dynamic network configurations. Therefore, the PLS approach can be used as an alternative supplement for computationally demanding high-layer technologies to further ensure data security.

Although the PLS can be precisely evaluated using popular performance metrics, such as secrecy capacity, secrecy rate, secrecy throughput, etc., which are discussed in detail in the literature, security performance is quantified by maximizing the performance difference between legitimate channels and wiretap channels [14,15,16,17]. This is intuitive since PLS aims to enhance the received signal quality at the intended receiver or reduce the performance of the wiretap channel relative to the legitimate channel. In this circumstance, there is a need to allocate transmission power based on the states of the legitimate and eavesdropper channels in order to improve the PLS, as transmission power affects the signal quality at the intended receiver and eavesdropper. However, the allocation of power in PLS is a challenging task. It relies heavily on the prior information that the transmitter has on the channel state information (CSI) of the intended receiver and the eavesdropper. Most of the optimization functions in PLS are non-convex because of the characteristics of the logarithmic subtraction in security performance metrics. For instance, when the transmission power increases, the capacity and reliability of the main channel improve [13]. On the other hand, the capacity of the eavesdropper channel may also improve and the probability of eavesdropping increases. Therefore, there is no universal approach to achieving a global optimization for non-convex power allocation. Several research works have been conducted to formulate and solve these optimization problems in order to obtain stronger security [18]. In [19], instead of maximizing the secrecy capacity of the main channel, suboptimal power allocation was presented to minimize the SINR at an unintended receiver. However, the minimization of the SINR at an unintended receiver is not assessed by direct performance metrics and the security measure cannot ensure a non-negative rate of transmission. Moreover, a joint subcarrier and power allocation mechanism were proposed in [20] for maximizing the secrecy capacity of OFDMA-based wireless networks. Nevertheless, the performance of secrecy gain can be enhanced by limited power allocation. Consequently, it is hard to achieve global quality of service (QoS) constraints for secure transmission systems.

The mainstream studies on PLS as a method for characterizing an achievable security performance against eavesdropping have been extensively investigated from the fundamental viewpoints of information theory for different communication scenarios and channel types and under different assumptions on the knowledge of CSI. These studies have inspired the development of many signal processing design techniques [21,22,23,24]. In this context, a large number of research works have been conducted, which have contributed insightful thoughts and opportunities to the understanding of practical security designs, optimization techniques, technology status, etc. For example, in [25,26,27], key technologies, technical challenges, and countermeasures were reviewed from the fundamental viewpoints of design strategies that involve physical-layer authentication, secret key generation, wiretap coding, and multi-antenna techniques, and relay cooperation. Moreover, the authors in [28,29] presented an extensive investigation of multi-antenna techniques in multi-user wireless networks using different assumptions on the availability of CSI. Providing security for multi-antenna techniques is an effective and powerful approach in PLS that can offer higher spatial degrees of freedom. The survey paper in [30] also provided a comprehensive overview of secure transmission designs from the viewpoints of information theory and optimization problems using security performance metrics. Furthermore, a comprehensive overview of fundamental classification and applications of existing PLS techniques was presented by [31]. On the other hand, the challenges that face PLS were reviewed in [32]. It can be seen that the hurdles facing PLS are issued from different assumptions regarding the characteristics of wireless channels and eavesdropper models.

Naturally, the concept of optimization techniques in PLS plays a pivotal role in practical security design and thus, has received considerable attention from the research community. In this review paper, due to the importance of secure transmission design in most practical scenarios, we were motivated to conduct a systematic overview of this research direction. It has to be noted that these studies have been extensively investigated and have been published in many PLS research works. Nevertheless, we outline a summary of some of the interesting studies in Table 1. In contrast to the aforementioned works, our review paper provides a brief overview of recent results and technical challenges for the system design and optimization techniques for 5G wireless networks. The main focus of this review paper is the existing techniques and design strategies for PLS optimization, optimization problems, and the solutions that are related to wireless PLS. Moreover, it inclusively discusses the applications of several enabling and computing technologies that could improve the corresponding research challenges. In order to address the limitations of existing optimization challenges, ML and AI technologies need to be efficiently integrated into 5G networks in order to produce better security and resource management. The use of ML and AI technologies within the field of mobile communication infrastructure has made significant progress in ensuring security, reliability, and resource allocations in a dynamic, robust and trustworthy way [33,34,35,36].

The contributions of this review paper are as follows. First, we introduced fundamental principles and different channel models. In this context, we briefly reviewed the common scenarios and limitations of different security methods to understand the basic theories that are related to PLS. Second, the information-theoretic security metrics and application scenarios in PLS were investigated to provide a brief insight into secure design strategies. Third, the potential research directions and technical challenges in PLS from the perspectives of security design and optimization approaches were widely discussed. Finally, we reviewed the potential advantages of using ML and AI enabling technologies to further improve conventional security technologies. In summary, this review paper identifies optimization challenges in terms of secure resource allocation and signal processing techniques and presents potential solutions through the introduction of AI- and ML-based technologies.

The structure of this review paper is outlined as follows. The abbreviations that are used throughout the paper are described in Table 2. Then, Section 2 introduces the fundamental concepts of PLS; specifically, the general system model, adversary model and different types of wiretap channel models. The performance metrics that are used in PLS to evaluate the security design strategies are discussed in Section 3. In Section 4, the research directions and technical challenges are investigated; specifically, the main issues and technical challenges of system design and optimization techniques, which are discussed from the viewpoints of secure resource management and signal processing techniques. Section 5 explains the paradigms of ML and AI within 5G networks and their applications in physical layer design and optimization. In Section 6, we present notable future directions and open challenges, followed by a conclusion in Section 7.

Note: In this paper, matrices are denoted by bold uppercase letters and vectors are denoted by bold lowercase letters. The Euclidean norm, transpose, conjugate transpose, conjugate transpose operation and mutual information are denoted by $\|.\;\|,\;{\left[.\right]}^{T},{\left[.\right]}^{H},\;{\left[.\right]}^{†}$ and I(.;.), respectively. Without the loss of generality, x represents the set of optimization variables.

## 2. Fundamental Concepts

**Figure 1:**
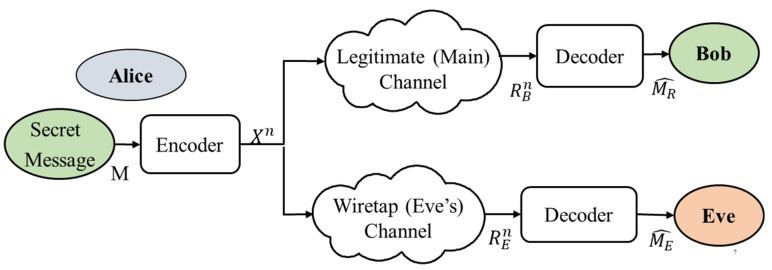
Generic system model of an Alice–Bob–Eve channel.

In this section, we provide insights into the general concepts and main preliminaries that are associated with PHY security. First, we provide a brief overview of the basic system model for PLS that is related to eavesdropping problems, as illustrated in Figure 1. Then, we consider and discuss the hurdles of PLS from the perspective of the adversary model that could prevent it from succeeding. The last part of this section presents several wiretap channel models that adopt the common notations and scenarios that are considered in the rest of the discussion.

### 2.1. Generic System Model

The typical network is where problems with PLS arise, which consists of a transmitter, a legitimate (intended) receiver and a malicious eavesdropper, represented by Alice, Bob and Eve, respectively. Figure 1 shows the general Alice–Bob–Eve model of a PLS system, in which the transmitter and receiver communicate through the legitimate channel and the eavesdropper makes a limited set of observations through a wiretap channel [37]. In this setup, the eavesdropper aims to decode or obtain sensitive information from historical observations of received signals. In other words, the objective of Alice is to use a secure transmission strategy that can send a confidential message to Bob while making sure that the eavesdropper is kept ignorant and is not able to glean any useful information from the transmitted signals. In order to achieve security in this case, PLS techniques are appropriately designed by exploiting the channel characteristics, such as noise, dispersion, fading, interference, etc., along with efficient secure transmission strategies, such that the information being sent from source to receiver is kept confidential from both active and passive eavesdroppers.

As illustrated in Figure 1, the transmitted message signal M is encoded into X with a length n and transmitted via a wireless medium. The signals that are received by Eve and Bob are designated by $R_{E}$ and $R_{B}$, respectively. The entropy function of the transmitting signal is denoted by H(M), while the residual uncertainty of Eve’s observations is given by $H\left(M/R_{E}\right).$ Taking the environment and scenarios into consideration, the availability of CSI at Alice, Bob and Eve varies from full or partial channel knowledge to zero information. The a priori knowledge that is available to Alice regarding the channel information of the legitimate and eavesdropper channels is crucial for the determination of the corresponding secrecy optimization scheme and PLS design. Nevertheless, in practical scenarios, it is logical to assume that the Alice is aware of the statistical information of the eavesdropper but not of its instantaneous realizations. Such information includes the type of channel distribution, the average gain of the received signal, the spatial direction and the line-of-sight (LOS) component.

### 2.2. Wireless Adversary Models

Due to the inherent characteristics of wireless communication, such as broadcast nature, openness and the superposition of the transmission medium, wireless networks are extremely vulnerable to security attacks. Within the field of security, an adversary refers to a wireless attacker who aims to disrupt or prevent transmitted signals from reaching the intended receiver. Therefore, it is important to consider adversaries when designing a secure strategy in order to measure the effectiveness of the proposed security system. Security attacks on wireless networks can be mainly classified into two types: active attacks and passive attacks [38]. The concept of passive or active attacks is typically to do with whether the adversary is actively injecting into the communication system or is merely listening to the ongoing transmissions [39]. Due to the various types of adversaries and the vast nature of attacks, there is a need to identify the assumptions, goals and capabilities on which these different types of PLS are designed and the potential challenges that could prevent PLS from succeeding.

A relevant set of goals is important for the modeling of a rigorous and strong adversary model. Naturally, a stronger adversary, i.e., with prior knowledge or more resources, could be able to attack the wireless network more successfully. With the adversary model, the assumptions of the adversary include its environment and resources, such as competency, knowledge, equipment, devices, etc. The adversary aims to compromise and obtain secret data content from within the communication system. These sets of data can be the legitimate transmission data or the property of the authorized user, e.g., energy consumption [40,41]. Due to their exposure to various types of attacks, communication channels are required to have determined capacities that enable them to resist and alleviate wireless attacks. These capabilities provide the adversary with reliable interactions that are based on the context of secure transmission systems. In fact, many of the PLS approaches assume that Alice has no knowledge of the eavesdropper’s CSI as the adversary is passive (i.e., not actively modifying the data, just silently reading and observing the communication system). On the other hand, some studies have found that Alice is sometimes assumed to know about the CSI of the eavesdropper [42,43,44]. Furthermore, active attacks have sometimes been observed, such as the denial of service, replay and node malfunction attacks that are employed against PHY security approaches. As a security community, we need to adopt a strong adversary model in which the adversary is cleverer and more active. Under the implications of an adversary being an active attack, intruders use more intrusive and aggressive methods that aim to deteriorate the received signal quality for the intended user.

The essential characteristics of a secure transmission system include authentication, availability, integrity, access control, and secret dissemination [38]. These can be established through appropriately designed signal processing strategies and channel coding techniques. The adversary model that is used in most PLS approaches is different from that used by the traditional cryptography and security community. Therefore, for the purpose of addressing the challenges that face PLS, it is important to bridge the gaps between the various adversary models that are used by the different communities. In the current review paper, the PLS mainly focuses on the premise that the eavesdropper is passive, i.e., it does not communicate with the other nodes in order to conceal its presence.

### 2.3. Wiretap Channel Models

The secrecy problem of a physical layer system involves the transmission of information signals through the legitimate channel without conveying the information via the wiretap channel. The PLS achieves perfect secrecy by exploiting an advantage of a legitimate transmission channel in the presence of an eavesdropper. Therefore, the wiretap channel model is a significant representative design that examines the relationship between the physical layer secrecy and channel capacity within a wireless transmission system. In general, the wiretap channel model consists of MIMO channels, multiple-access channels, multi-user broadcast channels and other channels, such as relay channels, interference channels, etc. [39].

#### 2.3.1. MIMO Wiretap Channels

A MIMO network structure consists of multiple transmit and receive antennae that allow for simultaneous secure broadcasts with less inference and noise. Providing physical layer secrecy via adaptations to the multi-antenna technique is a typical and essential type of security method, which can be applied in practical wireless communication systems [45,46,47].

**Figure 2:**
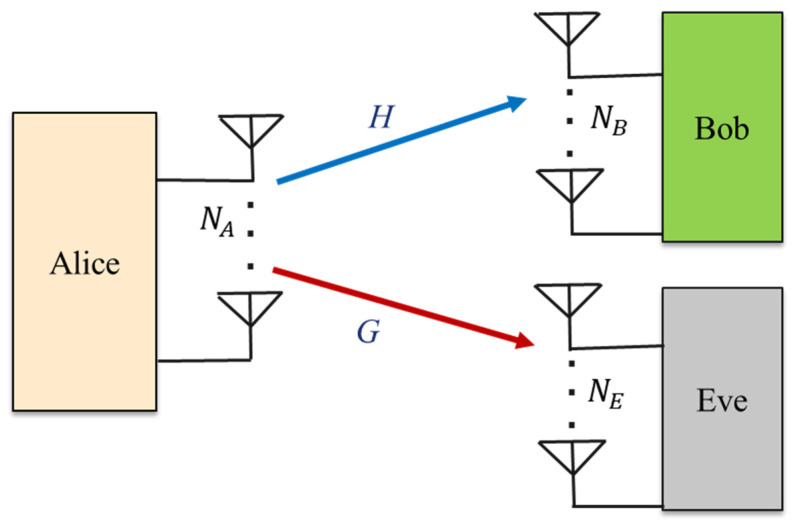
A system model for a MIMO wiretap channel.

In such multi-antenna scenarios, a typical MIMO wiretap channel model includes SISO, SIMO and MISO channels. In this review paper, we consider the more general setting that was studied in [39], in which the transmitter (Alice) wants to reveal the secret information to the intended receiver (Bob) over the main channel while the eavesdropper (Eve) acquires a noisy version of the transmitted signal via the wiretap channel, as shown in Figure 2.

In this setup, the Alice, Bob and Eve are equipped with multi-antenna systems, which are denoted by $N_{A},\;N_{B}\;and\;N_{E}$, respectively. Furthermore, the CSI of the main transmission and eavesdropper channels are denoted by ***H*** and ***G***, respectively. The general representations of the signals that are received by Bob and Eve, respectively, are described by the following equations:

(1) $$y_{B}=Hx_{s}+n_{B}$$

(2) $$y_{E}=Gx_{s}+n_{E}$$

where $x_{s}$ is the $n_{A}\times 1$ transmitted signal with a covariance matrix of $Q_{X}=E\left[x_{s}x_{s}{}^{H}\right]$ for $Q_{X}>0$. $H\in {∁}^{N_{B}\times N_{A}}$ and $G\in {∁}^{N_{E}\times N_{A}}$ are the MIMO channel matrices from Alice to Bob and Eve, respectively. Since the channels are discrete memoryless channels at the transmitter, each element of H and G obey the complex Gaussian distribution with channel gain coefficients of $h^{ij}$ and $g^{ij}$ between the *i^th^* source antenna and the *j^th^* intended receiver and eavesdropper antennae. The noise vector of the received signal antennae is assumed to be a complex Gaussian noise vector, with each element of $n_{B}$ and $n_{E}$ designed as an independent and identically distributed (IID) complex noise vector with the variance of $\sigma_{H}^{2}$ and $\sigma_{G}^{2}$, where $h^{ij}\sim ∁N\left(0,\sigma_{H}^{2}\;\right)$ and $g^{ij}\sim ∁N\left(0,\;\sigma_{G}^{2}\right)$.

It assumed that $n_{B}\in {∁}^{N_{B}\times 1}$ and $n_{E}\in {∁}^{N_{E}\times 1}$ are zero-mean AWGN vectors at the legitimate receiver, as well as at an eavesdropper, with $n_{B}\sim ∁N\left(0,\;I\right)$∼*CN(0,I)* and $n_{E}\sim ∁N\left(0,\;I\right)$, respectively. The maximum power of the transmitted signal from Alice is assumed to be P, where $E\left\{||x_{s}||{}^{2}\right\}\leq P$ for a total power P. Initiatively, Equations (1) and (2) are used as a basic optimization tool in PLS. Hence, the typical MIMO wiretap channel model has been widely considered in PLS to ensure secure data transmission.

#### 2.3.2. Broadcast Wiretap Channels

**Figure 3:**
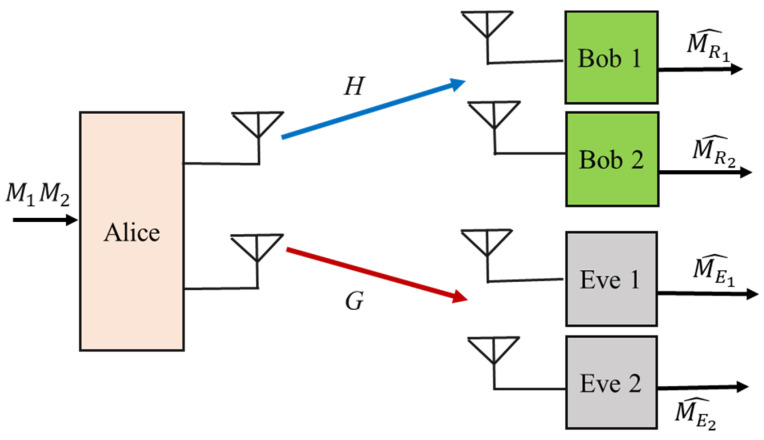
A system model for a broadcast wiretap channel.

In this channel model, Alice simultaneously transmits secret data content to multiple receivers in the presence of one or more unintended users, as shown in Figure 3. The common scenario for broadcast channels is a multi-user cellular network, in which the legitimate transmitter communicates with multiple intended receivers while being protected from eavesdroppers in the downlink. It is assumed that one source Alice is equipped with $N_{A}$ antennae and the intended I users and J eavesdroppers have $N_{Bi}\;\mathrm{and}\;N_{Ej}$ antennae, respectively.

In such scenarios, the signals that are received by other users may interfere with the desired signal due to the conditions under which each receiver obtains the mixed signals from all users. Furthermore, the susceptibility of eavesdroppers may also increase due to the opportunity for more information leakages in the downlink. Indeed, secrecy performance is not only affected by the eavesdropper and the mixed signals but also by the signals that are received by the other users [48]. Hence, a broadcast channel can be defined as a mixed multi-channel, which is described by [49,50] as follows:

(3) $$y_{Bi}=H_{i}x_{s}+n_{Bi},for\;i=1,2,\ldots ,\;I,$$

(4) $$y_{Ej}=G_{j}x_{s}+n_{Ej}\;for\;j=1,2,\ldots ,\;J,$$

where $x_{s}$ is the $n_{A}\times 1$ transmitted signal for the confidential information with a covariance matrix of $Q_{X}=E\left[x_{s}x_{s}{}^{H}\right]$ for $Q_{X}>0$. $y_{Bi}$ and $y_{Ej}$ are the signals that are received by the *i^th^* intended user and *j^th^* eavesdropper, respectively. $n_{Bi}$ and $n_{Ej}$ are the complex noise vectors at Bob and Eve, respectively. $H_{i}\in {∁}^{N_{Bi}\times N_{A}}$ and $G_{j}\in {∁}^{N_{Ej}\times N_{A}}$ are the matrices for channels ***H*** and ***G*** from Alice to the intended receiver *i* and unintended user *j*, respectively. Compared to the traditional MIMO channel system, the design techniques for secure transmission over broadcast channels are somewhat complicated. This is because the information leakages to eavesdroppers and the inter-user interference need to be mitigated simultaneously.

#### 2.3.3. Multiple-Access Wiretap Channels

**Figure 4:**
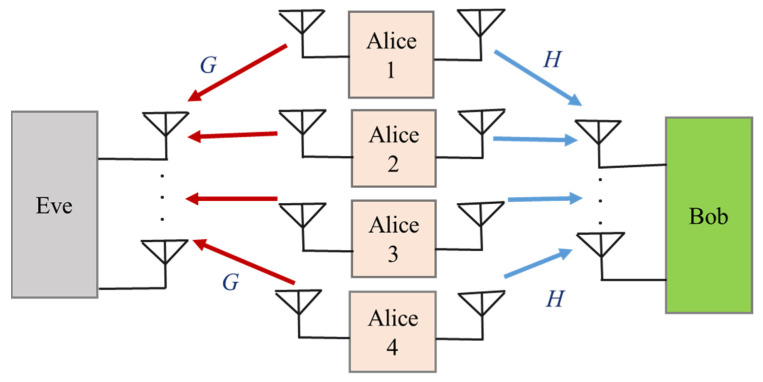
A system model for a multiple-access channel.

Multiple-access channels are another important form of multi-user network in which more than one legitimate source sends information to a common destination user in the presence of eavesdroppers that try to intercept and obtain the transmitted signals, as illustrated in Figure 4.

In this model, the transmitters are required to cooperate in order to guarantee secure transmission over the multi-access channel. However, due to the geographic isolation, the transmitters need to cooperate in a distributive manner. It is assumed that *k* transmitters are equipped with $N_{Ak}$ antennae and that one intended user and one eavesdropper have $N_{B}\;\mathrm{and}\;N_{E}$ antennae, respectively. Thus, the signals that are received by the intended user and an eavesdropper, respectively, are described as [51]:

(5) $$y_{B}=\overset{k}{\underset{k=1}{\sum }}H_{k}x_{s}{}_{k}+n_{B}$$

(6) $$y_{E}=\overset{k}{\underset{k=1}{\sum }}G_{k}x_{s}{}_{k}+n_{E}$$

where $x_{s}{}_{k}$ is the $n_{A}{}_{k}\times 1$ transmitted signal with an average power constraint or a covariance matrix constraint. $H_{k}\in {∁}^{N_{B}\times N_{A}{}_{k}}$ and $G_{k}\in {∁}^{N_{E}\times N_{A}{}_{k}}$ are the channel matrices from Alice to the intended user and unintended user, respectively. $n_{E}$ and $n_{B}$ are the AWGN vectors at the eavesdropper and intended receiver, respectively. In this survey, the secure multiple-access scenario is investigated from the fundamental viewpoints of information-theoretic security.

#### 2.3.4. Interference Wiretap Channels

**Figure 5:**
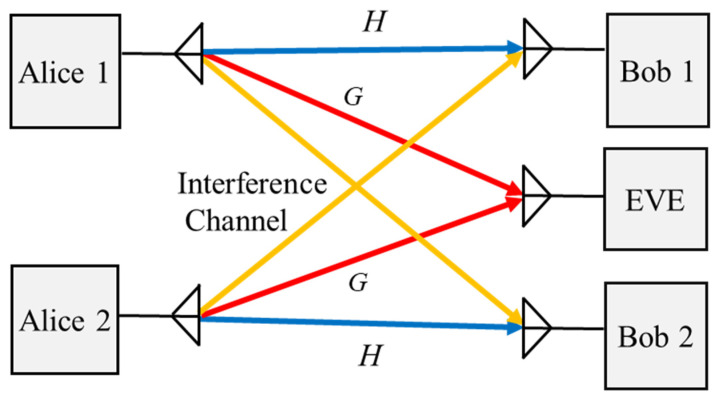
Secure communication using an interference channel model.

An interference channel is one of the channel models that are used in PLS, in which multiple legitimate parties (i.e., Alice and Bob) communicate with each other at the same time and using the same channel [52], as illustrated in Figure 5. At the same time, the communication between Alice and Bob is observed and decoded by an eavesdropper. Since all of the legitimate users are in the broadcast domain of service, there is a high probability of information leakage within this communication system. In order to avoid leakages to intended users and to enhance the security performance of the system, several multi-antenna techniques have been studied in the existing literature, such as interference cancellation and mitigation techniques [53,54]. It is assumed that the channel model with *K* transmitter and receiver pairs communicates with unintended users, where Alice *k* is equipped with $N_{A}{}_{k}$ source antennae and the destination receiver and unintended user each have receiving antennae of $N_{B}{}_{k}$ and $N_{E}$, respectively. Hence, the signals that are received by the intended user and Eve, respectively, are given by [27]:

(7) $$y_{B}{}_{k}=H_{k}x_{s}{}_{k}+\overset{k}{\underset{i\neq k}{\sum }}H_{ki}x_{s}{}_{ki}+n_{B}{}_{k}$$

(8) $$y_{E}=\overset{k}{\underset{i=1}{\sum }}G_{i}x_{s}{}_{i}+n_{E}$$

where $x_{s}{}_{i}$ is the $N_{A}{}_{i}$ × 1 encoded signal of Alice with an average power constraint or a covariance matrix constraint. $H_{ki}\in {∁}^{N_{B}{}_{k}\times N_{A}{}_{i}}$ and $G_{i}\in {∁}^{N_{E}\times N_{A}{}_{i}}$ are the channel matrices from Alice to the intended receiver and unintended user, respectively. $n_{E}$ and ${n_{B}}_{k}$ are the AWGN vectors at the eavesdropper and intended user, respectively. Furthermore, a typical configuration for interference channels with an external eavesdropper and an interference alignment was studied in [55]. It was shown that when an interference channel uses an interference alignment with separate confidential messages to reduce interference, the secrecy of the system can be improved. On the other hand, when an interference channel uses an interference alignment with an external eavesdropper, it lacks knowledge about the CSI of the eavesdropper and the secrecy of the system can be compromised. Therefore, the authors concluded that an interference alignment with separately secure information can provide more secure degrees of freedom than that with an unintended user.

#### 2.3.5. Relay Wiretap Channels

**Figure 6:**
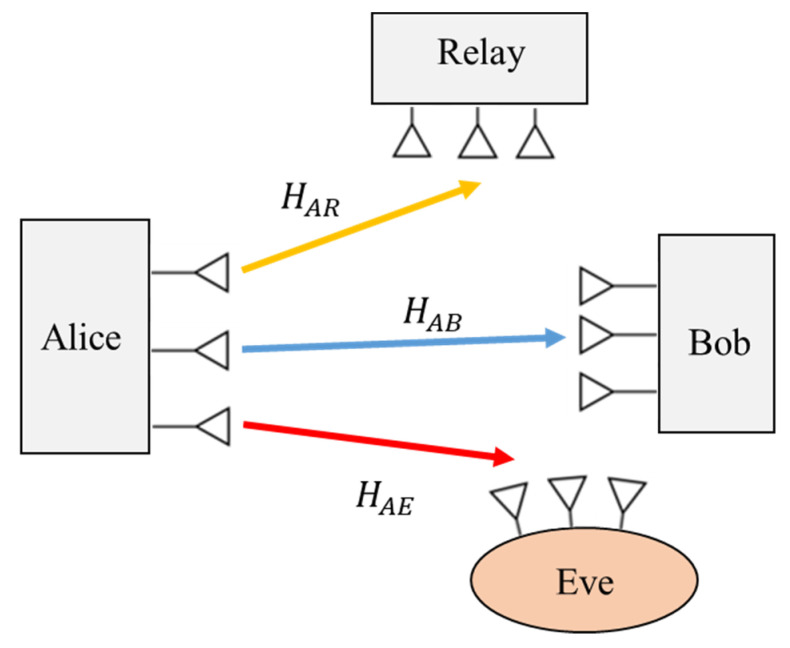
A system model for a wiretap relay channel in the first time slot.

**Figure 7:**
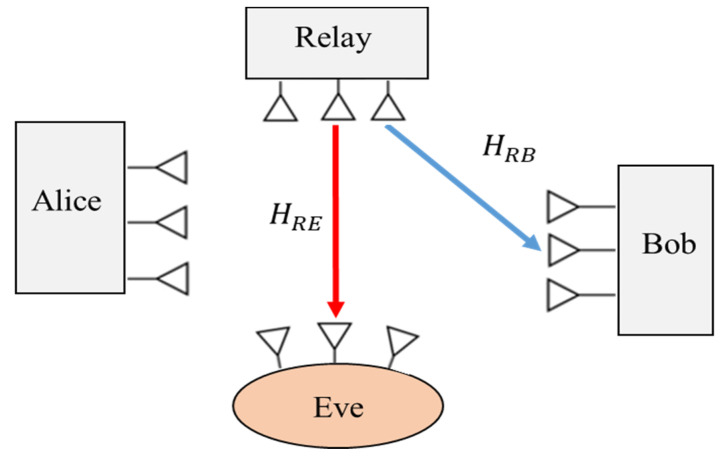
A system model for a wiretap relay channel in the second time slot.

Cooperative relaying is one of the multi-antenna techniques that have been presented to improve the PLS by increasing the quality of channel capacity between the source node and the destination receiver. In a secure relay network, the source and a relay node cooperate to improve the system security while preventing eavesdroppers. The two commonly adopted examples of relaying protocols are the AF (amplify-and-forward) [56,57,58] and DF (decode-and-forward) [59,60,61] schemes. Examples of typical relaying networks are shown in Figure 6 and Figure 7, which consist of a source (Alice), an intended receiver (Bob), the cooperative relay node and an eavesdropper (Eve). The relay node functions in a DF mode.

It is assumed that Alice, Bob, the relay node and Eve are equipped each with $N_{A},\;N_{B},\;N_{R}\;\mathrm{and}\;N_{E}$ antennae, respectively. In the first case, Alice sends the $N_{A}\times 1$ information signal vector $x_{s}$ to the relay node. Then, the received signals at Bob, the relay node and Eve, respectively, are given as [62]:

(9) $$y_{B\left(1\right)}=H_{AB}x_{s}+n_{B}$$

(10) $$y_{R}=H_{AR}x_{s}+n_{R}$$

(11) $$y_{E\left(1\right)}=H_{AE}x_{s}+n_{E}$$

where $H_{AB}\in {∁}^{N_{B}\times N_{A}}$, $H_{AR}\in {∁}^{N_{R}\times N_{A}}$ and $H_{AE}\in {∁}^{N_{E}\times N_{A}}$ are the matrices from Alice to the Bob, the relay node and Eve, respectively. Obviously, $n_{B}$**,** $n_{R}$ and $n_{E}$ denote the AWGN vectors at Bob, the relay node and Eve, respectively. The relay node decodes the original message signal and forwards it on to Bob.

In the second case, the relay node transmits a new version of $x_{s}$ with a weighting vector $x_{r}\in {∁}^{N_{R}\times 1}$ of the encoded signal. Hence, the signals that are received at Bob and Eve are described as:

(12) $$y_{B\left(2\right)}=H_{RB}x_{r}+n_{B}$$

(13) $$y_{E\left(2\right)}=H_{RE}x_{r}+n_{E}$$

where $H_{RB}\in {∁}^{N_{B}\times N_{R}}$ and $H_{RE}\in {∁}^{N_{E}\times N_{R}}$ are the matrices from the relay node to Bob and Eve, respectively. Without loss of generality, we investigated the decode-and-forward (DF) cooperative channel model. Another type of cooperative relaying protocol is the AF channel scheme, which has a great impact on the capacity of physical layer secrecy. An extensive investigation of such an AF scheme for secure relay communication is presented in [63,64].

## 3. Performance Metrics and Application Scenarios in PLS

Physical layer secrecy ensures information confidentiality by enhancing the performance difference between the main transmission and the wiretap channels. The selection of appropriate metrics is essential for this approach to secure transmission design. One of the critical steps that must be executed after the design of secure physical layer transmission is the proper measurement of the level of security using the right metric. Therefore, it is essential to select suitable performance metrics that can quantify the relevant aspects. The performance evaluation must indicate the level of security that the wireless schemes or techniques can provide. However, various challenges are commonly being brought up by different performance requirements, especially when the sequence of channel coding has a finite block length [65].

In this section, we present the basic secrecy metrics that are usually adopted in the design of efficient secure transmission strategies. It needs to be noted that the performance metrics that are used in the literature mainly focus on keyless PLS technologies, which are termed SINR-based performance metrics. SINR-based metrics are used to measure the achievable performance of a secure design under confidentiality constraints, namely the metrics that are related to the secrecy capacity/rate, secrecy throughput, power/energy consumption, secrecy outage probability (SOP) and quality of service (QoS). To be more specific, security metrics, as mentioned above, are commonly taken as the design metrics (criteria) for the design and optimization of PLS paradigms.

### 3.1. Secrecy Capacity Metrics

The goal of secrecy metrics is to quantify and evaluate the security performance of a communication system or a user’s privacy under the consideration of a specific adversary. The limits of PLS are characterized by the channel secrecy capacity or the more general trade-off between the maximum transmission rate and the secrecy capacity equivocation rate [66,67,68]. In PLS, the secrecy rate is described as the data that are transmitted via the given transmission medium per second, which are available on Bob’s channel but are not decoded on the eavesdropper channel. More precisely, to assess the security performance of a system, the achievable secrecy rate and the Gaussian channel inputs have usually been considered in pioneering works [69]. Therefore, the secrecy rate can be defined as the performance difference between the capacities of the main transmission and the wiretap channel. Considering the PLS system model in Figure 1, the achievable rate of a transmission strategy is described as:

(14) $$R_{s}={\left[R_{B}-R_{E}\right]}^{+}$$

where the conjugate transpose ${\left[x\right]}^{+}≜max\left\{0,\;x\right\}$. $R_{B}$ and $R_{E}$ are the data transmission rates of the Bob and eavesdropper channels from the source Alice to the legitimate user and intruder, respectively. Basically, the rate of transmission $R_{s}$ can be improved using signal processing techniques and optimization approaches, which have been proven to be the lower bounds of security channel capacity [69]. In the actual design of secure transmission systems, a non-zero rate of transmission can be achieved using some multi-antenna techniques, such as secure precoding, beamforming, adaptive resource allocation, etc. Such techniques attempt to intentionally degrade the wiretap channel while enhancing the channel quality for the intended receiver.

On the other hand, the performance metrics that are sometimes referred to as the upper bounds of secrecy rate were defined by [70,71], which play a central role in PLS in terms of secrecy capacity. More accurately, they describe the maximum transmission rate at which confidential messages can be securely delivered to the intended user while preventing the eavesdropper from decoding any important information within the communication. The secrecy capacity of a wireless transmission is an essential theoretical tool for assessing the performance of practical PLS system designs. By examining a channel’s secrecy capacity and related features, information-theoretic security can provide common ground and guidance for the design of secure wireless transmission systems. To maximize performance capacity, the qualities of both legitimate and wiretap channels play a significant role in AWGN channels [72]. This can be realized by optimizing the optimal input probability distribution P(X) of mutual information [73], which can be expressed as:

(15) $$C_{s}=\underset{p\left(X\right)}{\max}\left(I\left(X;Y\right)-I\left(X;Z\right)\right)$$

where I(X;Y)=H(X)−H(X/Y) and I(X;Z)=H(X)−H(X/Z). X represents the channel inputs that are sent by source Alice and Y and Z are the channel outputs that are received and observed by Bob and the eavesdropper, respectively. It should be noted that wiretap channels are degraded; therefore, the corresponding signals of X, Y and Z form a Markov chain in any distribution of p(X) [67,70]. Based on [72], the secrecy capacity of an AWGN channel is defined as the difference between the capacities of the main transmission channel and the wiretap channel, which is mathematically given as:

(16) $$C_{s}=C_{B}-C_{E}$$

where $C_{B}$ and $C_{E}$ are the secrecy capacities of the legitimate and wiretap channels, respectively. In this case, it is possible to obtain a non-zero security capacity when the eavesdropper channel is downgraded relative to the Bob channel. Specifically, the intended receiver and eavesdropper channels are supposed to have variances of $\sigma_{B}{}^{2}$ and $\sigma_{E}{}^{2}$, respectively.

It should also be noted that the secrecy capacity and secrecy rate that are discussed above do not consider wireless fading channels. More precisely, it is assumed that Bob’s channel is more substantial than the eavesdropper channel. However, in fading channel environments, the channel gains for both legitimate and eavesdropper channels change randomly over time while remaining constant in each time slot [70]. When considering the features of fading channels, the average secrecy capacity must be assessed to improve the resulting security. Consequently, the secrecy rate or secrecy capacity is a suitable metric for these fading channel scenarios [66]. In order to assess the security within the actual transceiver design, an achievable ergodic secrecy rate was proposed in [69] as a security metric under the assumption of fading channels. When the transmitting power constraint is P, the attainable secrecy rate can be defined as the maximum gap between the secrecy rates of the main transmission and eavesdropper channels. When considering average power, the secrecy rate of a secure transmission system is described as:

(17) $$R_{S}={log}_{2}\left(1+\frac{H_{AB}\;P}{\sigma_{B}{}^{2}}\right)-log\left(1+\frac{H_{AE}P}{\;\sigma_{E}{}^{2}}\right)$$

where $\sigma_{B}{}^{2}$ and $\sigma_{E}{}^{2}$ are the variance of noise vectors in the Bob and eavesdropping channels, respectively. Therefore, the attainable rate of transmission is assumed to evaluate the security performance of the designs for efficient transmission strategies under the consideration of fading channels. However, the secrecy rate is different for different optimization and signal design techniques. In order to evaluate better secrecy rate performances in multi-antenna techniques, a novel approach to the optimization technique was discussed in [74]. Generally, the secrecy rate can be maximized through the allocation of power according to the knowledge of the CSI of the transmitter. Therefore, when the combinations of CSI are different at the source Alice, the attainable secrecy rate may be different.

In summary, the secrecy rate or secrecy capacity of an Alice–Bob–Eve channel is mainly calculated using the difference between the capacities of the legitimate and eavesdropper channels and the availability of the CSI of the transmitter. Although the secrecy rate and secrecy capacity are well established and commonly used in information-theoretic security, these metrics only reflect the achievable bounds of the random channel characteristics and do not indicate the real security performance in practical design scenarios with various service applications. In order to measure and improve the resulting secrecy in fading channel environments, these metrics are extended to outage secrecy rate probability and outage secrecy capacity [75].

### 3.2. Secrecy Outage Probability (SOP)

A secure transmission system may be affected by imperfect CSI and wireless fading channels. Therefore, an appropriate secrecy metric needs to be adopted from the perspective of system design and signal processing approaches, which is referred to as secrecy outage probability (SOP). The SOP mainly focuses on the difference between the error probability rates of the legitimate user and the eavesdropper in order to achieve a practical security performance. More precisely, it describes the probability that a detailed evaluation of the secrecy rate for a specific secure transmission system is not achievable. This metric specifically investigates the information security and reliability of data transmission. Despite the importance of the conventional rate outage probability in characterizing and evaluating the achievable secrecy performance of fading channels, the SOP has three main weaknesses [76]: (1) the SOP cannot provide any insights into the capability of an eavesdropper to correctly identify secret messages; (2) the SOP cannot investigate the amount of sensitive data that have been revealed to unintended users when a secrecy outage event occurs; (3) the SOP cannot be related to the requirements of the quality of service (QoS) for different application scenarios. Analogous to the traditional rate outage probability, the SOP is used to estimate the probability that the instantaneous secrecy capacity $C_{s}$ is below than the actual secrecy rate $R_{s}^{0}$ [77,78,79,80]. The SOP of this definition is expressed as:

(18) $$P_{out}\;(R_{s})=P_{r}\{C_{s}<R_{s}^{0}\}$$

More specifically, the outage secrecy incidents $\left\{C_{s}<R_{s}^{0}\right\}$ in (18) indicate that the rate of transmission cannot be executed by the main transmission channels and that the secure communication would not be achieved. In this case, secure transmission would only be achieved when the main transmission channel has a greater SNR than the wiretap channel. On the other hand, the authors in [81] studied the SOPs and outage secrecy rates of quasi-static flat fading channels. They showed that secure transmission can be ensured even when the average SNR of the main transmission channel is less than the eavesdropper channel. Furthermore, their results demonstrated that the instantaneous secrecy performance of fading channel features would be greater than the secrecy performance of a non-fading channel with the same average SNR. Basically, the instantaneous rate capacity is different for the different realizations of fading channel scenarios. More precisely, the average secrecy capacity is equal to the maximum instantaneous secrecy capacity for fading channels. In order to maximize the secrecy performance of secure transmission strategies, the transmission power is optimally allocated based on the statistical distribution of Bob’s channel and the instantaneous realization of the eavesdropper channel. Mathematically, this can be defined as:

(19) $$max\left\{C_{B}-E\left[C_{E}\right]\right\};\;s.t.\;0\leq \emptyset \leq 1$$

where ∅ represents the ratio of power distribution across the main transmission channel and $E\left[C_{E}\right]$ is the expected capacity of the eavesdropper channel. The average secrecy capacity ($\bar{C_{s}}$) can be expressed as:

(20) $$\bar{C_{s}}=\bar{C_{B}-E\left[C_{E}\right]}$$

To be more specific, the $\bar{C_{s}}$ can be maximized through the allocation of power based on the assumptions on the knowledge of CSI at the source node [82]. Therefore, the $\bar{C_{s}}$ may be different when a source Alice has different knowledge about the CSI of the legitimate channels. More generally, it has been shown that the CSI of a legitimate channel is more important than the CSI of an Eve channel. An Alice can decide to send confidential information when the condition of the legitimate channel is strong enough and when the CSI is available at the transmitter. In this case, the CSI of the wiretap channel may not be important for secrecy capacity. For example, when a source Alice only has the CSI of the legitimate channel and not the CSI of the wiretap channel, the allocation of power can be executed according to the information of the CSI of the legitimate channel. Thus, it is possible to minimize the SOP by carefully modeling the achievable rate of the secrecy capacity $R_{S}$, the secrecy capacity of the legitimate channel $R_{B}$ and the conditions for secure transmission [83].

In summary, the secrecy outage probability (SOP) is used in cases where the source transmitter has very little information about the CSI of the intended receiver or the eavesdropper. The SOP is more applicable in conditions where the statistical CSI of a wiretap channel is known to the source Alice. In general, the secrecy outage capacity/probability is used to measure the reliability of secure transmission strategies.

### 3.3. Quality of Service (QoS)-Related Metrics

Compared to existing wireless communication networks, 5G and B5G networks have very high data rates and higher coverage rates with significantly improved QoS. One of the main demerits of employing traditional secrecy outage probability-based metrics for 5G networks is that they are not directly linked to the QoS requirements for different service applications. Without loss of generality, the QoS-related metrics that are used in this survey include SINR-based, PER-based and BER-based metrics. The SINR-based metrics are the most widely used metrics for describing the performance difference between the capacities of Bob and Eve channels. The theoretical basis of the SINR-based metrics can be characterized as the quantitative relationship between the noise powers of the legitimate channel and the Eve channel [29,30]. Additionally, the performance of the main transmission and wiretap channels is also described by the QoS. Thus, the SINR-based metrics are instantly linked to the QoS and this can help with the optimization and modeling of secure transmission strategies. A maximum SINR performance of communication from the source Alice to Eve and a minimum SINR performance from Alice to Bob imply that the system performance for receiving signals is good enough in terms of the reliability and security aspects. This includes the method for efficient secure communication that is capable of obtaining the required maximum and minimum error levels for Eve and Bob, respectively. In order to enhance the capacity of a transmission link, it is important to adopt PLS techniques, such as beamforming. An example of this definition is the packet error rate (PER) [84] and bit error rate (BER) [85], which can be analogously related to secrecy throughput [86] and, therefore, the SINR. For the foundation of a transmission channel, the BER requirement must be greater than the minimum desired level. In general, the physical layer techniques exploit optimization problems to enhance the security of a transmission strategy by reducing the BER of eavesdroppers. As a result, the BER can be utilized to estimate the performance of a secure transmission strategy, as well as the QoS requirements.

Furthermore, the authors of [87] provided an alternative design for a secure precoding scheme, which was based on the QoS and SINR metrics. This design contains an optimal power allocation and standardization framework schemes, which also realize the trade-off between minimizing SINR at the eavesdropper and maximizing SINR at the legitimate receiver. Despite the existing knowledge-rich approaches, secure and reliable transmission strategies are still a predominantly open problem within physical layer design and optimization systems. In these cases, the appropriate metrics are commonly used during security design to estimate the attainable performance of transmission systems. Specifically, the secrecy metrics that are listed in Table 3 are mostly considered to be the design criteria and optimization constraints that are necessary for efficient secure communication strategies.

## 4. Research Directions for System Designs and Optimization Concepts

Mainstream studies on PLS system design can be generally summarized by two main approaches. The first is related to secrecy features, which particularly focuses on the characterization of security capacity and eavesdropping or the more general trade-off between attainable secrecy capacity and confidentiality equivocation based on the concepts of information-theoretic fundamentals. The second approach is related to secure system design, which mainly focuses on the design and optimization of secure transmission strategies through the use of signal processing techniques [37,69,94,95].

**Figure 8:**
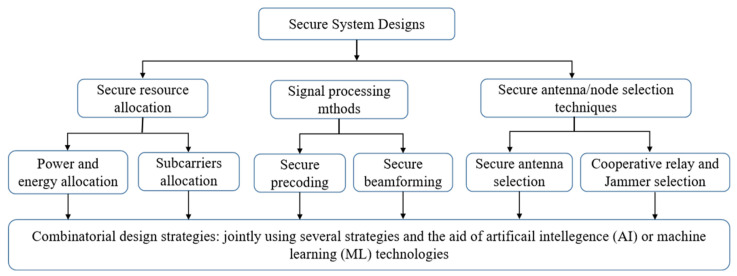
Secure transmission strategies to improve the security and robustness of physical layer designs.

Many conventional technologies in physical layer security, without the consideration of secrecy communication, can be reconstructed for secure data transmission under the fundamental framework of PLS. To realize the basic optimization problems and performance metrics of PLS, the main issues and research directions are expected to include three candidates for secure design strategies, as illustrated in Figure 8. These research topics are signal processing techniques, secure resource allocation and secure antenna selection/cooperative nodes. Improvements in secret communication within PLS can be supported by these research topics [30]. The signal processing techniques utilize secure precoding and beamforming to achieve the design of efficient secure communication strategies. The secure precoding and beamforming designs fully utilize the characteristics of multi-node and multi-antenna scenarios, which may form virtual or massive MIMO networks. Resource allocation, which is usually adopted within conventional communication systems, includes the allocation of power and subcarriers. It mainly focuses on resource management systems that utilize multi-faceted wireless resources, including power, time slots and frequency. Cooperative nodes or secure antenna selection, such as jammer selection, relay node selection and user selection, which are widely used in multiple node scenarios, have been fully explored as promising methods to improve the design of PLS. Such techniques attempt to select appropriate cooperative nodes or antennae from a candidate set to enhance the efficiency of secure design strategies.

Despite the unparalleled advantages of the research approaches that are mentioned above, it is worth noting that some drawbacks do exist. To achieve a fine-grained security performance, only using a single research approach may be difficult or even insufficient for future wireless systems. Therefore, the joint use of some of the above techniques that is based on several enabling technologies would be more efficient for ensuring the security of the whole transmission system [34]. Typical examples include joint user scheduling and resource allocation and the trade-off between reliability, security performance, latency, energy consumption, etc. Furthermore, considering joint strategies, examples of the application of AI and ML technologies in physical layer security design and optimization are discussed in detail in Section 5.

### 4.1. Main Technical Challenges in System Design

**Figure 9:**
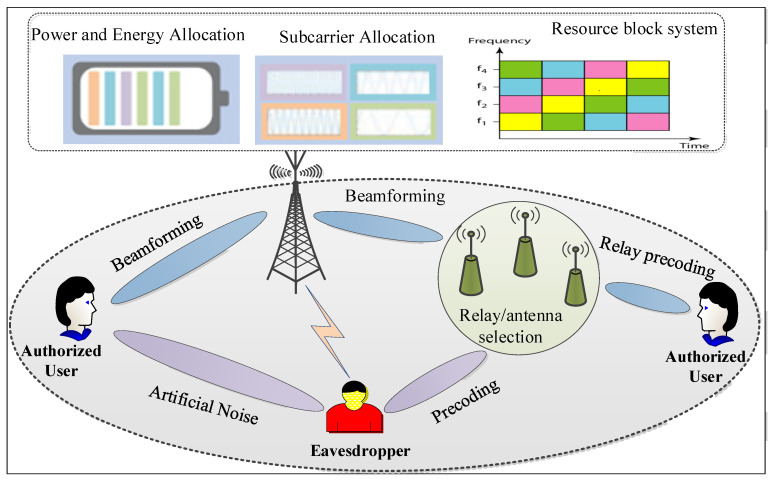
An illustration of multi-dimensional security and resource management within a multi-service wireless network.

Keyless-based PLS techniques are well established transmission strategies that can enhance the performance variation between the main transmission channel and wiretap channels. Unlike conventional transmission methods, the optimization objectives, the conditions of constrained optimization problems and the performance parameters that are associated with SINR-based security techniques are based on the characteristics of wireless channels and information theory secrecy metrics. For secure physical layer design and optimization schemes, the selection of suitable performance metrics is crucial. As illustrated in Figure 9, multi-dimensional security and resource management strategies typically contain secure resource allocation and signal processing methods for cooperative wireless networks. Resource allocation systems are employed by the network operator, which are shown in the top position; simultaneously, the signal processing schemes are operated by transmitters, which are positioned on the bottom. Multiple-antenna techniques have been widely used in wireless networks due to their spatial degrees of freedom, which are offered by multi-service networks. Although secure multi-antenna techniques are widely implemented in various design approaches, they can also be mathematically modeled as optimization problems to find the most favorable transmission solutions. This can be realized and optimized by using information-theoretic security metrics to design secure beamforming and precoding, appropriate antenna or relay node selection and resource allocation. Most of the problems in PLS are caused by non-convex optimization due to the characteristics of quadratic programming functions in the performance metrics. Many researchers have carried out extensive work on formulating and solving these optimization issues in order to obtain the maximum level of security [96]. By considering the complexity of these non-convex constraint functions, several signal processing techniques and optimization methods have been developed to solve the corresponding optimization problems.

Although many optimization methods have been developed to cope with the non-convexity of the optimization problems in PLS schemes, there are still many challenges to solve within the existing schemes; specifically, the basic assumptions that are related to system design and channel coding models. Given the limited resources in wireless networks, such as energy and bandwidth, a common problem that is associated with resource management is the adequate exploitation of the resource constraints to attain the conditions of the information-theoretic security metrics. In a transmission system with limited resources, design and optimization schemes must be considered for the resource allocation to the various consumers who use the data over the network so as to achieve a good performance. Several existing works have discussed the fundamental resource management issues within multi-dimensional wireless networks, such as power allocation, subcarrier allocation and joint power and subcarrier allocation [97,98,99,100,101,102,103,104,105,106]. The existing secure resource allocation methods are generally based on communication nodes with three terminals (i.e., the Alice–Bob–Eve model) and lack sufficient investigation into multi-user scenarios and heterogeneous networks. On the other hand, the existing design and optimization of signal processing schemes are commonly based on the parallel fading channel and AWGN, with few investigations on massive MIMO and millimeter wave channels. Therefore, the PLS is still a great potential prospect for 5G wireless communication systems to mitigate all of these challenges. As discussed above, the complexity of networks and channels can affect the corresponding optimization objectives, which results in additional non-convex optimization problems that are hard to address. The major challenge in the existing signal processing methods is the computational cost of implementing optimal secure precoding and beamforming schemes in practice. When considering the challenges and problems that are associated with system design, potential research directions and corresponding enhancement schemes can be identified and evaluated by reaping the benefits of ML and AI enabling technologies, which are discussed in detail in Section 6.

### 4.2. Optimization in PHY Security: Current Status and Main Issues

The information-theoretic studies on the investigation and characterization of attainable rates of security performance for multi-dimensional secure wireless networks that are presented in [31,81,107,108] have inspired the optimization and system design of many signal processing techniques. From the fundamental viewpoints of optimization objectives, four representative techniques have been adopted in this area: (1) convex optimization; (2) secure beamforming; (3) artificial noise (AN); and (4) zero-forcing (ZF) precoding.

#### 4.2.1. Convex Optimization Techniques

Convex optimization has been widely used in PLS because it can effectively evaluate information-theoretic security metrics to find the most favorable transmission solutions. In PLS, the optimization problem refers to the problem of determining the performance metrics, such as power consumption, secrecy outage probability, secrecy capacity or secrecy rate, etc., which were discussed in Section 3. In the existing studies on PLS, there are some convex optimization techniques that are widely used to resolve optimization problems, such as in [109]. As mentioned earlier, due to the properties of the logarithmic subtraction functions in the performance metrics, most of the optimization issues in PLS are either non-convex or quadratic programming functions. These non-convex optimizations are adopted to solve problems that are related to non-linear constraints and objective functions. Therefore, non-convex optimization problems can be used in the design of complex global and optimal solutions. Some common examples of non-convex optimization problems include secure power allocation, power minimization and beamforming. In order to achieve optimal solutions, the original non-convex problems need to be converted into convex problems using other functions, such as SDP. Semi-definite programming (SDP) can be used to optimize a linear constraint function under non-negative and linear equality constraints. In general, in order to solve the problems of non-convexity optimization in PLS system design, many optimization methods have been discussed in the literature, such as semi-definite relaxation (SDR), alternating search, dual decomposition, etc. [37,97,110].

#### 4.2.2. Secure Beamforming Techniques

The basic idea of beamforming is to transmit signals effectively in the direction of Bob and Eve, which aims to enhance the signal quality of the main transmission channel and degrade the quality of wiretap channels. Beamforming is a typical signal processing method that has been shown to be a promising means to achieve PLS [95,111,112,113]. The optimization concern of secure beamforming-based designs is to reduce the interference signal at the intended receiver so that the desired recipient can receive the desired QoS via the transmitted signal. For secure beamforming, the optimization problems are neither concave nor convex due to the characteristics of the logarithmic functions in the performance metrics. Due to this, optimization problems are solved using basic numerical calculations in many situations, for example, as in [63,90,114,115,116]. However, the complexity of the numerical methods is computationally prohibitive, which affects the design of secure beamforming within practical applications. In order to reduce the complexity, some low-computational algorithms have been explored in the literature, which facilitate practical application scenarios, such as in [117,118,119,120,121,122]. Therefore, due to their simple design schemes, the developed algorithms are attractive alternatives that attempt to maximize secrecy performance and, at the same time, minimize interference to the secure transmission.

#### 4.2.3. Artificial Noise (AN) Techniques

AN is an effective technique for multi-antenna systems and has been extensively considered within PLS in order to enhance communication security even further. The essence of the AN technique is to generate noise in the null space of the main transmission channel; simultaneously, it is also designed within the range of the wiretap channel [85]. The basic idea is to generate an AN signal that is based on the assumptions of the eavesdropper so that the intended receiver is not affected. This technique aims to degrade the quality of the unintended receiver’s channel by adding a noisy signal, which is used to interrupt their attacking abilities [123,124,125]. On the other hand, it is designed to avoid interference leakage for the intended user. In multi-antenna scenarios, it is possible to adjust the directions of the transmit and AN signal jointly through spatial beamforming by exploiting the degrees of freedom to optimize the performance metrics. In practice, performance of the AN technique depends on the knowledge of the CSI of the eavesdropper at the transmitter. Naturally, knowledge of the full CSI of both the wiretap and legitimate channels is important for practical security design and optimization schemes to attain the optimal performance of secure transmission. When the CSI of the Eve channel is known at the source Alice, the maximum spatial degrees of freedom are applicable, i.e., the beamformer is perfectly designed. However, according to practical scenarios, the CSI of the eavesdropper is usually either imperfect or unknown. Several methods have been proposed in the literature to deal with these assumptions, such as those that are presented in [126,127]. They provide the optimal power allocation and secure beamforming scheme between AN and transmit signals in order to maximize the achievable secrecy performance. The major strength of the AN design is that the offered secrecy performance matches the SNR well because when the SNR increases at the eavesdropper, the power of the noise signal increases, along with the transmitting power.

#### 4.2.4. Zero-Forcing (ZF) Precoding Techniques

Precoding is another signal processing method that is used to deliver signals to the null space of wiretap channels. In ZF precoding schemes, the source Alice that uses multiple transmit antennae is based on the cancelation of unwanted signals at the destination node in multi-user or multi-stream data transmission [128]. Some authors provided an iterative algorithm to control the interference signals that are leaked to unauthorized users. The design of secure precoding that is based on ZF beamforming was discussed in [129] and requires both the complete CSI of the main transmission and wiretap channels at the transmitter and that the number of intended user antennae is greater than that of the eavesdropper. To be more specific, the security performance of ZF precoding becomes very poor when knowledge of the CSI of the eavesdropper at the transmitter is limited, which makes it less applicable.

In summary, the concepts of optimization objectives, constrained conditions and corresponding performance parameters were discussed based on four basic signal processing techniques in order to easily understand design and optimization problems. Although optimization problems that use convex optimization achieve better secrecy performance than the conventional secure beamforming and zero-forcing precoding techniques, convex optimization is more complex and expensive to apply to different services within practical scenarios. To tackle these network complexity and computational costs, service providers should develop solutions to ensure reliability and provide secure resource management to their customers in a robust, intelligent and reliable way. In the next section, we discuss the use of AI and ML technologies as key enablers for achieving the above-mentioned system performance metrics.

## 5. Paradigms of AI for Physical Layer Optimization and System Design

In the face of new service requirements and heterogeneous networks, it is challenging to meet the existing physical layer design and optimization techniques through the use of one method alone. In order to solve the more complex optimization problems in PLS, many enabling technologies will be used in B5G communication networks. As powerful tools and intelligent problem-solving algorithms, ML and AI can be widely applied in the design and optimization of physical layer security paradigms. In this section, examples of ML- and AI-based solutions in physical layer design and optimization are discussed, including point-to-point multi-antenna techniques, secure resource allocation and signal processing methods.

### 5.1. Overview of AI and ML Enabling Technologies

As powerful and intelligent tools, AI and ML can be widely applied in the optimization and system design of 5G network infrastructures in order to solve complex problems [130,131,132]. AI and ML approaches have been widely used to solve complex problems within the upper layers of open system interconnection models, such as the deployment of wireless communication and cognitive radio relay networks, which produce significant performance improvements compared to secure wireless communication systems that were designed using conventional methods [133]. However, considering the recent success of current trends and the challenges of future wireless communication systems, such as high-speed connection demands in complex scenarios with unknown channel models, AI and ML approaches have now also been considered in the physical layer of wireless transmission strategies [134,135,136,137]. Recent advances in AI and ML techniques, especially deep learning (DL), reinforcement learning and convolutional neural networks (CNN), have provided novel concepts and potential opportunities to solve these complex problems. Extensive research has been conducted on the application of ML- and AI-based methods in 5G and B5G networks, for example, as presented in [138,139,140,141,142,143]. Examples of typical applications in the physical and data link layers include the use of DL or reinforcement learning algorithms to evaluate and predict channel quality, the use of orthogonal frequency division multiplexing (OFDM) for symbol detection at the receiving end, channel encoding and decoding and dynamic spectrum random access, among other functions. In channel quality assessment, deep neural network (DNN) algorithms analyze limited pilot signals to help massive MIMO systems infer complete and accurate knowledge of CSI. Therefore, performance enhancement, quality of service and low computational costs can be achieved by applying CNN, DNN and reinforcement learning algorithms in channel encoding and decoding.

Furthermore, AI learning-based methods have been used to design universal frameworks for various technical challenges for base stations, characteristics and applications, which have contributed to the enhancement of 5G wireless networks. AI algorithms can be implemented to address various problems in resource utilization, network traffic and user demand. They can make it possible to smartly coordinate 5G base stations (BSs) and other core 5G network elements, such as user plane functions [144,145,146]. ML and AI schemes can help to further improve reliability, security performance, energy efficiency and network latency by developing integrated scheduling and efficient allocation schemes. Considering the potential of PLS in the system design and optimization of 5G networks, the challenges and corresponding opportunities for intelligent AI and ML technologies to achieve a high level of security at the physical layer, as well as cross-layer applications, deserve to attract more attention from the research community. AI and ML algorithms are suitable for the analysis of future 5G networks and the adaption of resource management and network protocols for different applications. These algorithms have their own different pros and cons. An overview of some of the notable techniques and focused issues that are associated with AI and ML technologies are presented in Table 4.

In the next subsections, we discuss some ML- and AI-based applications in the design and optimization of 5G networks, which cover three different problems within the physical layer of wireless networks, including secure multi-antenna techniques, secure resource management and the optimization of signal processing methods.

### 5.2. AI for End-to-End Multi-Antenna Techniques

In conventional physical layer modules, secure transmission design usually relies on non-interactive random-coding arguments and mainly focuses on the establishment of theoretical achievements. In this case, it is possible to achieve a good security performance by demonstrating the existence of PLS codes, such as low-density parity-check (LDPC) codes, lattice codes and polar codes. However, the design of explicit security using these conventional methods is less reliable in practical and realistic communication systems [151]. To be more specific, the optimization problems of the physical layer of beyond 5G networks are non-deterministic polynomial (NP) problems and are becoming more challenging as there is a series of barriers between different signal processing blocks, such as non-orthogonal multiple-access (NOMA) channel encoding and signal detection, the space–time processing of multiple user MIMOs and channel decoding for polar or LDPC codes [152,153]. In such scenarios, LDPC codes have a higher decoding complexity in large blocks or under poor noise conditions, while polar codes require multiple iteration convergences to achieve optimal performance. 

In order to cope with the existing gaps, AI and ML techniques have been applied to simplify and handle physical layer modules [154,155,156,157]. Specifically, deep neural network (DNN) algorithms have been widely implemented in various signal processing function blocks, such as MIMO detection [158], polar code decoders [139], modulation recognition [159], etc. AI can be mainly applied to CSI processing, receiver design and end-to-end link design at the physical layer. For example, neural networks in deep learning have been used to learn the compressed representations of high-dimensional CSI in wireless communication, thereby reducing the CSI feedback overhead. For the physical layer modules, there have been many related AI-based works that have addressed the design and optimization problems of end-to-end multi-antenna systems. In [160], DNN was employed for a joint optimization scheme in end-to-end communication. In contrast to conventional channel equalizer and channel decoder approaches that follow non-interactive schemes, the DNN-based joint optimization consists of an adaptive learning (encoding) module and an end-to-end communication module, which are interactive. This approach is very useful for reducing the computational cost of training and online tasks.

**Figure 10:**
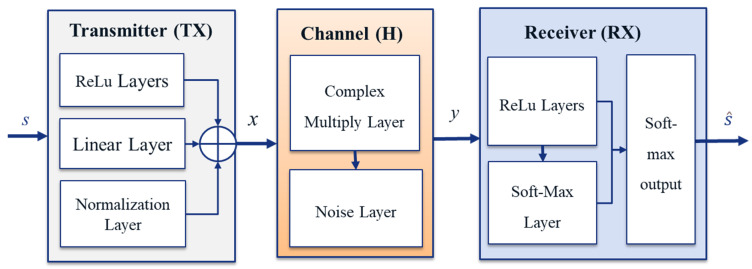
An illustration of a MIMO channel autoencoder.

Furthermore, an ANN-based autoencoder was proposed in [161] to generate end-to-end system optimization, which comprises a model-based solution and adaptive learning modules that can obtain important information in real-time scenarios. This framework has established applications in multi-antenna MIMO channel systems, which consist of three functional blocks: the transmitter, the MIMO channel generator and the receiver, as illustrated in Figure 10. The results showed that the learning module aims to encode the data inputs in an unsupervised fashion, which has the ability to redesign sets of data inputs at the output module. The functionality of the autoencoder is obtained by iteratively training the transmitter and receiver modules with block error rate (BLER), which is represented as fully connected DNNs. The resulting system has advantages for guaranteeing energy constraints. This scheme was later extended by other researchers to include multiple user MIMOs [162] in order to achieve better enhanced BER performances in the MIMO channels. This functionality is performed by learning from the input data and training for different scenarios with multiple antennae and various levels of CSI knowledge.

Therefore, existing optimization problems have proved the advantages of using AI, specifically DNN-based schemes for end-to-end communication without the assumption of several models in the existing conventional design concepts. Thanks to the potential of autoencoders, the complicated issues that cannot be modeled by conventional methods can be solved by training the scenarios and then optimizing the performance.

### 5.3. Applications of AI in Secure Resource Allocation

Resource management is a crucial issue for 5G networks, including inter-cell resource block allocation, computing power and energy allocation, available spectrum and communication channels, massive MIMO user clustering and beamforming resource allocation in distributed network structures [163,164,165]. Secure resource allocation in B5G networks is a more challenging and multi-objective performance optimization problem. The essential task of resource allocation in 5G networks is conducted to predict future service requests, mobility and the location of users. Since the emergence of new technologies and services in 5G that typically have limited power and restricted processing and are delay-sensitive, it has become useful to match these multi-objective requirements with minimum valuable resource costs, such as power and energy. The existing secure resource management and utilization schemes that have been widely adopted in traditional design concepts are based on end-to-end performance metrics, with less focus on multi-dimensional wireless networks. This requires the help of AI and other intelligent computing technologies to overcome these gaps in the existing schemes. The application of AI and ML techniques has great potential in these areas.

**Figure 11:**
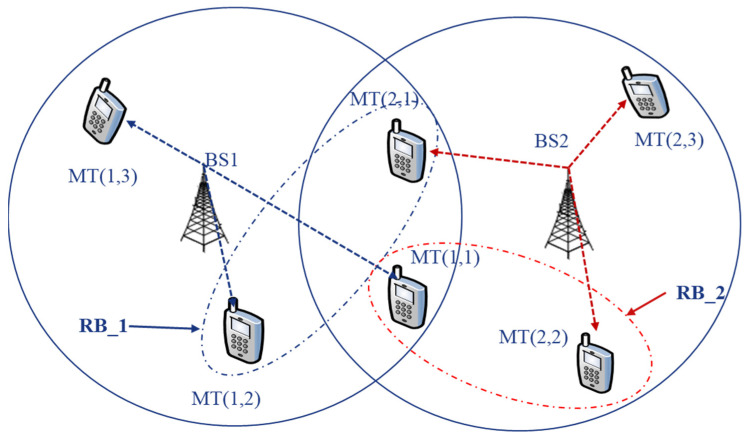
An illustration of downlink resource allocation for a multi-service network.

In this subsection, we consider typical multi-dimensional downlink resource allocation in a multi-cell, multi-user OFDMA-based scenario, as shown in Figure 11. In this framework, since the OFDM resource block (RB) distribution to various users within the same cell is orthogonal, the intra-cell interference signals are removed over wireless channels. The detection of OFDM symbols at the receiver usually depends on a receiver that uses maximum throughput estimation for evaluation, but this method is very sensitive to CSI errors and the accuracy of the model itself. The design of RB allocation for users is mostly carried out using non-linear programming, which involves non-deterministic polynomial (NP) optimization problems [153]. Nevertheless, the high complexity of this optimization method is prohibitively expensive; hence, it is hard to apply this method in practical scenarios. Therefore, it is evident that the traditional method for reducing computational cost is becoming less reliable. By applying AI to this NP problem, resource allocation can be further improved compared to existing resource management schemes. More specifically, reinforcement learning approaches (e.g., the Q-learning algorithm) can help to allocate resources efficiently.

The Q-learning algorithm was designed with the goal of maximizing the entire system performance by avoiding the allocation of similar RBs to neighboring cells that are located close to boundaries. The main motivation for applying Q-learning to RB allocation in downlink OFDM systems was discussed in [153]. According to [149], the Q-learning scheme can solve the NP combinatorial optimization problems that are hard to handle in the existing static partitioning of networks. Intuitively, the Q-learning algorithm allows an agent to interactively learn the optimal allocation policy by sharing resources with neighboring cells.

The main strategies include: (1) the allocation of free RBs to users in the same cell by assigning higher SIRs to the users; (2) the interactive updates of the allocated RBs for each user, based on the Bellman equation [166], in order to improve the system throughput performance; (3) the avoidance of allocating the same RB that has been assigned to users in neighboring cells. Consequently, by employing the Q-learning algorithm, a system can maximize its overall capacity by adjusting the RB allocation of each user. Additionally, the Q-learning approach promotes the fair allocation of user power since the involved users cannot acquire essential SIR from neighboring cells. Furthermore, a generalized Nash equilibrium framework was proposed in [167] to provide power optimization for users from multi-cells that are allocated to the same RB. Moreover, to design efficient resource allocation policies for 5G, the possible applications of AI were discussed in [168,169].

### 5.4. AI for Signal Processing Design

One important study on PLS system design was issued to explore optimization techniques and signal processing design fundamentals. The signal processing methods in 5G networks, such as secure precoding and beamforming, have been utilized to enhance various performance requirements in secure transmission systems. By optimizing these techniques, the appropriate performance metrics of PLS can be achieved while the desired QoS can also be ensured at the same time [170,171,172,173,174,175]. However, due to the complexity of the hardware and mathematical models in these performance metrics, the optimization problems of existing signal processing schemes become more and more complicated for future network environments [64,90,114,115,116], which means that: (1) it is hard to solve these problems using numerical methods because they are too sophisticated and have many imperfections in practical applications; (2) in order to obtain optimal design schemes, the hurdles between various signal processing barriers need to be reduced using optimal solutions; (3) the vastly increasing complexity of the systems and hardware installation that are needed to mitigate computational cost requires robust and intelligent algorithms to make system design more practical.

To cope with these challenges, the assistance of modern computing technologies has been used as a performance metric. On the other hand, ML and AI techniques can benefit from the optimization of traditional signal processing techniques. Some of the application examples include secure physical layer modules [176,177], channel estimation and equalization [178], obstacle detection and localization [179,180], modulation recognition [159] and channel coding and estimation [181,182]. The advantages of employing AI and ML technologies in 5G networks compared to traditional signal processing methods are two-fold. First, AI and ML approaches are designed to achieve optimal end-to-end performance metrics, while conventional schemes are performed by logically discrete blocks that are separately optimized. Second, artificial neural networks (ANNs) are known to be highly reliable and self-adaptive and have also been shown to have computationally universal functions [183]. ANNs are used to learn inverse mapping from received interference signals to the original signals without the need for explicit channel estimation and equalization. The joint optimization of transmitters and receivers within specific channel environments can produce non-ideal effects in the wireless channel and consequently, transmission performance is enhanced. By applying ANNs in signal processing schemes, we can improve optimal performance metrics as long as a reliable hardware implementation model exists. In summary, ML- and AI-based algorithms are more efficient and powerful for hardware implementation and have extensive applications that are not limited to signal processing schemes.

## 6. Discussions on Future Research Directions and Challenges

In the previous sections, it was shown that PLS has received significant research interest within the fields of information-theoretic fundamentals and signal processing. However, future wireless networks will become increasingly heterogeneous and will have new features and more complex network environments. Even though the research community has made great efforts in the existing works, many technical challenges still exist, namely the basic assumptions regarding channel types, eavesdropper models, the availability of CSI and application scenarios. Most of the existing assumptions that are used in PLS may be insufficient or unreliable for future wireless communication in practical security systems. Hence, there are still many technical challenges that need to be discussed in order to facilitate the integration of PLS into practical design systems. In the following section, we discuss some future directions and notable challenges, which are based on the knowledge that has been gained from the existing literature. It should be noted that only a few research directions are discussed in this section because the future work regarding PLS is a pervasive field of study.

### 6.1. The Joint Optimization of Reliability, Security and Resource Allocation

An optimal global design for system security, reliability and secure resource allocation is an important research idea that has not yet been well discussed in the literature. Newly introduced features and technologies in the core 5G network make the design of secure transmission approaches necessary and challenging. To enhance the optimal adaptability of user experience and network performance in wireless transmission systems, the reliability, security and allocation of necessary resources should be considered jointly in secure system design [12,184]. However, in many existing studies, except for some studies that have investigated the trade-off between energy consumption and security performance, most methods have been considered separately and individually to minimize the complexity of security design [9]. Therefore, achieving a joint optimization between reliability, security performance and resource consumption with low latency is a challenging problem. In this case, when the three optimization factors interact with each other, the proposed security mechanisms become potentially optimal and trustworthy. In order to achieve global optimization performance, some researchers [34] proposed an AI-based integrated scheduling system that can realize an optimal power allocation scheme and improve the security and reliability of legitimate transmission. The proposed scheme was designed based on the integration of AI and edge computing to reduce the computation latency within transmission channels. The proposed framework can be applied to the PLS paradigm to design signal processing techniques and secure resource allocation schemes. However, the energy dynamics within the storage capacities of smartphone devices and the distributed resource management in edge cloud computing make this framework challenging to offload tasks for users. Therefore, this area of research is still in its infancy and many issues need to be investigated in future research.

### 6.2. The Joint Design of Classic Cryptographic and PHY Securities

Another promising and futuristic research topic, which has not yet been investigated deeply in the literature, is the joint design of PLS and classic cryptographic techniques. The upcoming B5G networks require high security to ensure the most secure service applications. High-layer cryptographic techniques, such as privacy-preserving, authentication and encryption, will still be the main safeguarding mechanisms in data privacy and network security for B5G. Such techniques are usually implemented at the higher layers of data processing stacks. However, the advancements in wireless networks and the limitations of energy consumption in the mobile devices, hardware and processing capability in B5G networks require lower cost and more efficient data privacy and network security solutions. Dynamic and robust security solutions, such as secure service-oriented and group-based authentication systems [185,186], will be the major future directions for classic cryptographic security and privacy. In addition to the upper layer cryptographic techniques, the PLS is required to offer another layer for safeguarding data confidentiality in B5G networks [186,187,188]. PLS is emerging as a promising technique for managing the high costs of computational complexity and the resource consumption problems. Nowadays, PLS techniques have been widely adopted to provide secure transmission in emerging technologies, such as massive MIMO, advanced channel coding, terahertz (THz), millimeter wave (mmWave), etc. However, employing only PLS techniques may not be sufficient to ensure efficient and robust security in future 5G and 6G networks. PLS can be integrated into classic cryptographic techniques to improve the requirements of future wireless communication systems. Therefore, the basic issue is the joint utilization of the benefits of PLS and classic cryptographic security techniques.

To this end, cross-layer security design approaches aim to integrate higher layer security and PLS to provide secure strategies for each layer of data processing protocol. This can be performed by utilizing the mechanisms and functionalities of the two layers for security purposes. In order to achieve this challenging task, there are still many issues that are expected to be solved in future research. For example, secure coding techniques, network protocols, adaptive modulation, hybrid encryption algorithms and other techniques can be adopted as joint mechanisms for security. However, the structures of B5G networks are typically decentralized and transmission nodes are expanded with different characteristics. This requires a joint security scheme to adopt the same variety of nodes and heterogeneous architecture of B5G networks. In order to satisfy the requirements of service-related and user-related elements in B5G networks, dynamic, robust and efficient secure joint security techniques need to be developed [186].

### 6.3. The Impacts of ML on Channel Estimation

Channel state information (CSI) has a significant impact on the design of secure transmission strategies. However, the availability of CSI at the communication entities differs from full knowledge or partial knowledge to zero information. The acquisition of the CSI of an eavesdropper for the transmitter is not a simple task in actual security design. Due to the hidden nature of eavesdroppers and CSI feedback, it is difficult to obtain the CSI of wiretap channels at the transmitter. On the other hand, the CSI of legitimate channels can be obtained through CSI feedback from the legitimate receiver in FDD systems. To obtain the optimal performance of secure communication, correct channel estimation is indispensable for guaranteeing the quality of service (QoS). Nevertheless, it is not easy to obtain perfect CSI due to the limited feedback and estimation errors. In many situations, CSI can be imperfect or even unknown. Therefore, the challenges that are raised by channel estimation aspects are due to the difficulties in the perfect channel estimation of eavesdroppers and the considerations of CSI feedback delay, node mobility and channel correlations.

Recently, there have been plenty of efforts to use ML and DL techniques in channel estimation [189,190,191,192]. Based on these investigations, issues regarding the estimation of the proper channel of an eavesdropper and the tackling of imperfect CSI in system design still remain. It has been proven that the existing channel estimation schemes that are based on channel modeling are insufficient for obtaining perfect and timely CSI. By introducing ML techniques into channel estimations, the performances of existing channel estimation techniques can be improved, with low complexity in the practical applications [193]. Furthermore, DL has been employed for channel estimation in mmWave massive MIMO systems [194]. By applying intelligent denoising-based message passing techniques, the system can learn channel structures from training datasets and estimate accurate channels. In [195], an off-grid channel estimation algorithm was proposed for orthogonal time frequency space (OTFS) systems. This algorithm can adopt the sparse Bayesian learning model to accurately exploit channel sparsity. From the above discussions, the development of generalized ML- and DL-based channel estimation schemes for different scenarios remains a major challenge for future research. In order to design these generalized schemes, a large number of training datasets need to be used by the DL and ML algorithms to learn channel features.

### 6.4. The Influences of AI and ML on B5G Security and Privacy

The advent of new network features, technologies and services in B5G networks will reveal potential security and privacy challenges. These challenges are not only from the perspective of the consumer but also from the viewpoints of vendors, network operators and service providers in terms of developing perfect deployment of service scenarios. Service providers should produce solutions to ensure the security, privacy and allocation of essential resources to their customers robustly and reliably in order to tackle these challenges. Enabling technologies, such as AI and ML, are integrated into 5G and B5G networks and are expected to meet the security and privacy requirements to a certain extent. However, the adoption of AI and ML technologies in wireless networks will raise concerns about privacy and security issues, such as those raised in [196,197,198,199]. In order to address these problems in B5G networks, proactive privacy and security measures need to be developed. Such techniques will employ novel AI technologies and algorithms and subsequently, physical layer security may become one of the major areas of application for AI [200]. Due to the increase in data rates in B5G networks, the processing performance of such network elements will be further expanded, which will facilitate the strong combination of AI and future communication security design [201]. An ML-based autoencoder was proposed in [202] to reconstruct detection errors in order to discover irregular traffic data in future networks. ML-based PLS techniques, especially those regarding device authentication, were discussed in [203] to achieve a robust and highly reliable authentication for B5G networks. In [204], a big data analytics technique was applied to facilitate the rapid growth of B5G network data in specific scenarios. However, big data analytics can help to address abnormal user detection applications.

### 6.5. The Impacts of Adversarial Attack Models for Beamforming Systems

Adversarial attacks and adversary models are important issues that also need to be considered in secure physical layer transmission. In the existing research, most secure design strategies assume that adversarial attacks are passive. In this case, there is no cooperation or active exchange of information with other nodes within the system. However, in practical security design, there can be active cooperation and communication in confidential information exchanges [205]. Some strong adversaries may aim to estimate the environment in order to build up their advantages and threaten secure transmission [206,207]. Considering these observations, the design of secure strategies for PLS techniques face great challenges. One of the major challenges of this direction, specifically in beamforming systems, is information leakage into the subspace of eavesdroppers. The basic idea of beamforming is to transmit signals in the direction of legitimate channels. When transmission power is allocated between intended and unintended directions, most of the power is allocated to the main side lobes and a small portion of the power is allocated to the minor lobe beam. Transmitting signals are then leaked through the eavesdropper channel [113]. According to [125], beamforming techniques attempt to enhance the quality of signals at the main channel. In this case, the design of beamforming does not consider the possibility of eavesdroppers when its channel gain is higher than that of the main channel. Therefore, it is challenging to design a beamformer that is optimal for the legitimate user under the favorable channel settings of an eavesdropper. It is computationally expensive to achieve a perfect balance between optimal signal power and signal leakages. The challenges of the existing channel training and narrow beam scanning in multi-antenna techniques can be addressed through the use of ML and AI algorithms to achieve optimal solutions with less complexity [149]. Unfortunately, there are also potential security concerns in the integration of ML algorithms into future 5G technologies. A recent study on beamforming prediction tackled the security problems of using ML algorithms [51]. It was shown that in order to investigate beamforming prediction, adversarial attack strategies should be studied according to the loss maximization-based attacks that are relative to AI models.

## 7. Conclusions

Recently, PLS has attracted broad research interest in terms of the design of efficient and secure transmission strategies for 5G wireless networks. This review paper provided detailed discussions on the basic optimization schemes and system design of PLS, based on both information-theoretic security and signal processing fundamentals. To understand the advantages of PLS, we first provided a comparison between the traditional cryptographic and PLS techniques. Then, we introduced different wiretap channel models and their mathematical expressions in order to understand the common scenarios of practical security design. From the information-theoretic security perspective, we reviewed popular performance evaluation metrics, including the rate of transmission metrics, secrecy capacity, secrecy throughput, secrecy outage probability, secure resource allocation and QoS-related metrics. Then, we reviewed optimization problems and security solutions from the viewpoints of system design and signal processing methods, such as convex optimization, secure beamforming, artificial noise (AN) and zero-forcing (ZF) precoding. Regarding research directions, we discussed candidates for future research topics within PLS, including resource allocation, signal processing techniques, secure node/antenna selection and cooperative networks. Thereafter, AI and ML applications were reviewed to solve different problems for optimization and security design. Specifically, we discussed the paradigms of AI and ML in terms of some promising research directions, such as signal processing design, end-to-end multi-antenna techniques and OFDM-based RB allocation applications. We also discussed other research directions and the open challenges that face PLS in future wireless networks. In summary, our review incorporated different aspects that are related to the optimization and design of PLS systems, including secure resource allocation, channel estimation and the integration of AI into practical strategies to help PLS. We believe that this paper can provide the research community with guidance for advancing the considerations of future PLS research.

## Figures and Tables

**Table 1:** Summary of related studies.

Existing Papers	Research Issues	Important Content
[12]	Examine the security threats and corresponding defense methods in PHY security.	Summary of the security requirements and threats in wireless networks considering the network protocols at various levels of data layers. Additionally, a comprehensive review of state-of-the-art PHY security and existing security protocols for 13 various wireless networks.
[25]	A comprehensive survey on the basic theories and key technologies of PHY security.	Discussion of the key technologies, limitations, and solutions of PHY security from the perspective of security coding, physical-layer authentication, secret key generation, and multi-antenna techniques.
[26]	Security threats and the corresponding countermeasure techniques.	Technologies, security attacks, and defense mechanisms in PHY security using game-theoretic approaches.
[27]	Overview of the key technologies of PHY security.	Recent technologies, optimization techniques, and limitations of PHY security from the perspective of information-theoretic security and wiretap channels.
[28]	A comprehensive investigation of multi-antenna techniques.	Survey of multi-antenna techniques in multi-user networks for improving the security performance of PHY security, but not considering CSI accuracy.
[29]	A brief survey on multi-antenna techniques.	Investigation on multi-antenna techniques in PHY security for improving secrecy performance considering the accuracy of CSI.
[30]	A comprehensive overview of the optimization and design strategies of PHY security.	Survey on security designs and optimization techniques from the viewpoints of information theory and security metrics in wireless PHY security.
[31]	Comprehensive overview of all existing PHY security techniques.	Classification of the existing PHY security techniques and brief discussion of the big picture they can be easily understood and applied in different communication systems.
[32]	Challenges of PHY security in real-world systems.	Identification of the existing assumptions and opportunities for applying PHY security in practical applications.
[34]	A comprehensive investigation of AI and edge computing (EC) for PLS.	Identification of the existing challenges in the design and optimization of PLS and design of an enhancement scheme for PLS application.

**Table 2:** Abbreviations and their full form definitions.

Acronyms	Full-Form Definition
5G	Fifth-generation mobile networks
AI	Artificial intelligence
AF	Amplify-and-forward
ANN	Artificial neural networks
AWGN	Additive white Gaussian noise
B5G	Beyond fifth-generation networks
BER	Bit error rate
BLER	Block error rate
CNN	Convolutional neural network
CSI	Channel state information
DF	decode-and-forward
DL	Deep learning
DNN	Deep neural networks
FDD	Frequency division duplexing
IoT	Internet of Things
ILDP	Interactive learning design paradigm
LDPC	Low-density parity-check
LOS	Line of sight
MISO	Multiple-input single-output
MIMO	Multiple-input multiple-output
ML	Machine learning
mmWave	Millimeter wave
NOMA	Non-orthogonal multiple access
NDP	Non-deterministic polynomial
QoS	Quality of service
PLS	PHY security/Physical layer security
PER	Packet error rate
OFDM	Orthogonal frequency division multiplexing
SDP	Semi-definite programming
SOP	Secrecy outage probability
SIMO	Single-input multiple-output
SISO	Single-input single-output
SDP	Semi-definite programming
SNR	Signal-to-noise ratio
SIPNR	Signal-to-interference-plus-noise ratio
ReLU	Rectified linear units
ZF	Zero forcing

**Table 3:** Summary of performance metrics for PHY security.

Metric Types	Definition	Optimization Problems
Secrecy capacity	The maximum (upper bound) of the secrecy rate [72].	Transmission effectiveness of secure communication strategies.
Secrecy rate	The transmission rate that can be genuinely supported by the main transmission channel but not decoded on the eavesdropper channel [85].	
Secrecy outage probability (SOP)	The probability that the actual or targeted transmission rate is greater than the instantaneous secrecy capacity [86,87,88].	Reliability and security of communication systems.
Quality of service (QoS)	The performance improvement of secure transmission strategies, which includes the SINR-based, PER-based and BER-based metrics [27,31,89].	QoS and security of transmission systems.
Power/energy consumption	The minimum power consumption that is needed to ensure secure QoS requirements for different services [90,91,92,93].	Resource consumption costs for secrecy performance.

**Table 4:** Overview of ML and AI techniques for physical layer design and optimization.

ML- and AI-Based Techniques	Focused Issues	Advantages	Limitations
AI and edge computing (EC) [34]	Investigation of the gap between PHY security and AI–EC.	Robust PHY layer key generation schemes and secure resource management frameworks.	Complexity of training models for various PLS issues.
Deep reinforcement learning [35]	Enabling of secured visible light communication (VLC).	Achievement of the optimal solution between secrecy rate and utility.	Avoidance of the quantization error.
Integrated AI [36]	Integration of wireless power transfers and cooperative jamming for secure transmission.	Achievement of the trade-off between security performance and energy consumption.	Limitations of optimal scheme for solving more complex problems.
Iterative water-filling algorithm [147]	A comprehensive investigation of MIMO eigenmode transmission.	Bridging of the gap between AI and 5G technologies.	Challenging integration of AI and 5G networks.
Distributed AI federated learning [148]	A brief survey on multi-antenna techniques.	Contribution of robust and fine-grained security metrics.	Security issues at the device level.
Feed-forward DL model [149]	RF beamforming codeword prediction.	Promising results for beamforming problems.	Lack of investigation into security issues.
Adversarial DL model [150]	Adversarial attacks for beamforming prediction.	Consideration of security issues.	Complexity of adversarial training approach.

## Data Availability

Not applicable.
